# The Gutta Percha Tree

**Published:** 1864-05

**Authors:** 


### THE GUTTA PERCHA TREE.

The tree called the Isonandra Gutta, which furnishes the gutta percha, is a native of the Indian Archipelago and the adjacent islands. A few years since this substance, now of such widely extended use, was totally unknown in Europe, for though from time immemorial the Malays employed it for making the handles of their hatchets and creeses, it was only in the year 1843, that Mr. Montgomery, an English surgeon, having casually become acquainted with its valuable properties, sent an account of it, with samples, to the Royal Society, for which he received its gold medal. The fame of the new article spread rapidly throughout the world; science and speculation seized upon it with equal eagerness; it was immediately analyzed, studied, and tried in every possible way, so that it is now as well known and as extensively used as if it had been in our possession for centuries. The Isonandra Gutta is a large high tree, with a dense crown of rather small, dark-green leaves, and round smooth trunk. The white blossoms change into a sweet fruit, containing an oily substance fit for culinary use. The wood is soft, spongy, and contains longitudinal cavities filled with brown stripes of gutta percha. The original method of the Malays, for collecting the resin, consisted in felling the tree, which was then placed in a slanting position, so as to enable the exuding fluid to be collected in banana leaves. This barbarous proceeding, which, from the enormous demand which suddenly arose for the gutta, would soon have brought the rapidly rising trade to a suicidal end, fortunately became known before it was too late, and the resin is now gathered in the same manner as caoutchouc, by making incisions in the bark with a chopping-knife, collecting the thin white, milky fluid which exudes in large vessels, and allowing it to evaporate in the sun, or over the fire. The solid residuum which is the gutta percha of commerce, is finally softened in hot water, and pressed into the form of slabs or flat pieces, generally a foot broad, a foot and a half long, and three inches thick. Gutta percha has many properties in common with caoutchouc, being completely insoluble in water, tenacious but not elastic, and an extremely bad conductor of caloric and electricity. The uses of gutta percha are manifold. It serves for water-pipes, for vessels fit for the reception of alkaline or acid liquids which would corrode metal or wood, for surgical implements, for boxes, baskets, combs, and a variety of other articles.—*Hartwig's Tropical World.*

